# Vascularised and Non-Vascularised Adipofascial Flap Applications in Tissue Trauma with Tendon Injury, Flap Viability and Tendon Healing a Hystological and Scintigraphical Rat Model Study

**DOI:** 10.3390/medicina60101689

**Published:** 2024-10-15

**Authors:** Mehmet Yucens, Ahmet Nadir Aydemir, Tarık Sengoz, Gulcin Abban Mete, Nusret Ök, Mehmet Rauf Koç, Ahmet Fahir Demirkan

**Affiliations:** 1 Faculty Depth of Orthopaedics, Pamukkale University Medicine, Denizli 20160, Turkey; anaydemir@yahoo.co.uk (A.N.A.); nok@pau.edu.tr (N.Ö.); fahirdemirkan@yahoo.com (A.F.D.); 2 Faculty Depth of Nucleer Medicine, Pamukkale University Medicine, Denizli 20160, Turkey; tsengoz@pau.edu.tr; 3 Faculty Depth of Histology, Pamukakle University Medicine, Denizli 20160, Turkey; gabban@pau.edu.tr; 4 İzmir City Hospital, İzmir 35510, Turkey; mehmetraufk@hotmail.com

**Keywords:** tendon repair, adipofascial flaps, vascularised flaps, non-vascularised flaps, scintigraphy, histopathology

## Abstract

*Background and Objectives*: Complex wounds in the hand and distal lower extremities pose challenges in reconstructive surgery, often involving critical structures like tendons. Tendon injuries, prevalent in such wounds, necessitate optimal repair methods for functional recovery. This study investigates the impact of vascularised and nonvascularised adipofascial tissue on tendon repair, focusing on early healing stages, mobilisation, and scintigraphic evaluation of flap vascularity. *Materials and Methods:* Wistar Albino rats were divided into groups undergoing primary tendon repair, vascularised adipofascial flap application, or nonvascularised flap application. Scintigraphic evaluation and histopathological assessment were performed to analyse healing processes. *Results*: Pedicle-free flaps support healing in tendon injuries without negatively affecting medium-term outcomes. Vascularised flaps exhibit faster healing. The scintigraphic analysis showed that the static measurements of the late phase were statistically significantly higher in the group with the non-vascularised adipofascial flap (*p* = 0.038). The mean perfusion reserve was higher in the vascularised pedicled adipofascial flap group than the non-vascularised adipofascial flap group. Scintigraphic analysis highlights the viability of pedicle-free flaps. *Conclusions*: Pedicle-free adipofascial flaps support the healing of the tendon without complicating the results, while vascularised flaps show accelerated healing. These findings provide valuable insights into optimising tendon repair strategies using adipofascial flaps, with implications for enhancing functional recovery in complex wounds.

## 1. Introduction

Reconstructing complex wounds in the hand and the distal third of the lower leg, foot, and ankle presents a formidable task in the field of reconstructive surgery. These wounds are typified by the exposure of critical deep structures, including bone, joints, tendons, or fracture fixation implants. Additionally, they may involve chronic wound infections such as osteomyelitis, as well as complicating factors like smoking and medical comorbidities such as poorly controlled diabetes [1]. Tendon injuries are common soft tissue injuries among wound types. Primer tendon repair is the preferential treatment. However, the best treatments are controversial and range from “wait-and-see” approaches to surgery, with the appropriate treatment generally selected based on the type of injury. There is no clear consensus on how to perform tendon repair, especially in tendon injuries with a bad wound bed. The priorities of treatment methods are to close the wound and obtain a functional limb. For this reason, early rehabilitation and mobilisation are key objectives [2]. Adhesions and inadequate tendon healing can occur following tendon repairs, especially when the surrounding tissue is damaged. Even Müller et al. studied the effect of the paratenon and found that recovery of mechanical strength and tendon healing was higher in the paratenon intact group. They also found early collagen formation [3]. Various strategies have been employed to mitigate adhesion formation [4,5]. To obtain a functioning limb, the concept of interposing vascularised tissue, such as adipofascial flaps, between the tendon and the surrounding tissue has been utilised, particularly in the acute and subacute phases [6]. Sometimes adipofascial flaps may be required to provide tissue coverage for injuries with a poor wound bed or to prevent tendon adhesion and protect vascular nerve structures. It is not always possible to carry adipofascial flaps with appropriate vasculature. In cases where such vasculature cannot be found or preserved, a non-vascularised application may be an option. Adipofascial flaps serve as protective barriers for tendons, nerves and vascular structures in injuries with compromised wound beds in both the upper and lower extremities, and they are also utilised for staged wound closure [7,8]. Fascial flaps have emerged as the preferred choice for covering exposed bone, nerves, and tendons in hand injuries [9]. They have also been employed in the treatment of nerve tissues in patients with neuritis, utilising adipose tissue, fascia, or muscle transfer [10]. Adipofascial flaps or tendon repairing techniques can be used with or without additional treatment [11,12].

The aim of this study is to investigate the viability of vascularised and non-vascularised adipofascial flaps when applied over a repaired tendon and tendon healing.

## 2. Materials and Methods

### 2.1. Animals

In this study, experiments were performed on 18 male Wistar albino rats 200 ± 12 g at Pamukkale University Laboratory Animal Center. Ethical approval was obtained from Pamukkale University Laboratory Animal Ethics Committee (PAUHADYEK-2018/37). The rats were randomly divided into three groups with six rats in each group. Randomisation was performed using a computer-generated random number sequence to ensure unbiased allocation. The person responsible for generating the random sequence was not involved in the experimental procedures, thereby reducing the risk of allocation bias. After generating the randomisation sequence, rats were assigned to one of the following groups: Group 1 (primary tendon repair), Group 2 (adipofascial flap with a vascularised pedicled group), or Group 3 (adipofascial flap applied as a non-vascularised group). This random allocation was performed by an independent researcher who was not involved in the outcome assessment or data analysis, ensuring allocation concealment. Rats were housed under temperature-controlled conditions (20 ± 2 °C) in standardised cages with a 12-h light/dark cycle and ad libitum access to food and water. Prior to inclusion in the study, the animals were kept in the laboratory for at least one week to minimise stress. All experiments were conducted in accordance with IACUC guidelines. The study was documented in accordance with ARRIVE reporting guidelines [13], and power analysis, based on data from previous studies of rat tendon healing, determined that six tendons per treatment group per time period were required for 90% power [11,12].

### 2.2. Surgical Protocol

**Figure 1:**
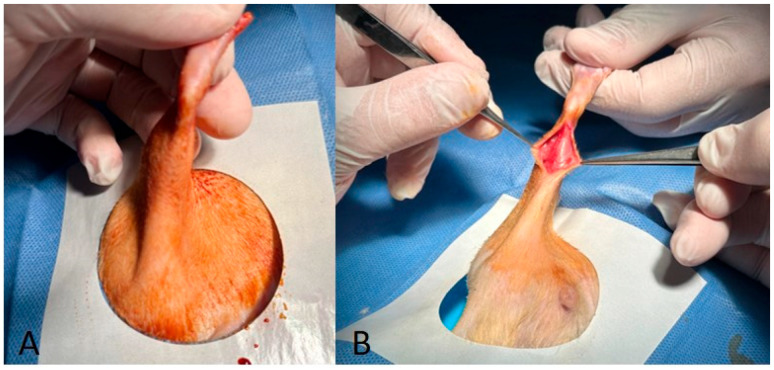
(**A**) Depilation and sterilisation with povidone iodine. (**B**) Precise incision the gastrocnemius fascia was released from proximal to distal.

Surgical anaesthesia was induced by intraperitoneal administration of ketamine (90 mg/kg body weight) and xylazine (10 mg/kg body weight). After induction, the right legs of each rat were carefully prepared by depilation and sterilisation with povidone iodine (Figure 1A). A precise incision was then made along the posterior aspect of the leg from the gastrocnemius muscle to the heel. (Figure 1B).

In Group 1, the gastrocnemius fascia and Achilles tendon were carefully identified, after which the Achilles tendon was transected using a scalpel and tendon repair was performed using a modified Kessler technique combined with an epitendinous continuous suture. Subcutaneous and skin closure was then performed sequentially.

**Figure 2:**
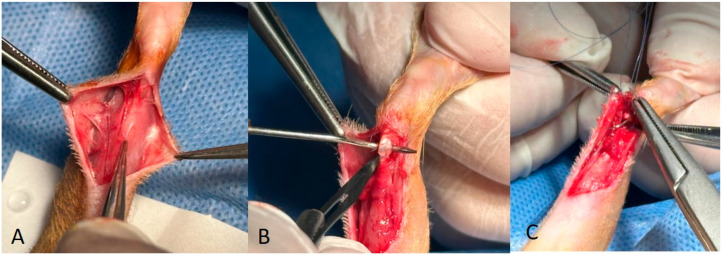
(**A**) The arterioles of the gastrocnemius fascia. (**B**) The Achilles tendon was cut transversely with a scalpel. (**C**) The Achilles tendon was repaired using a modified Kessler technique.

**Figure 3:**
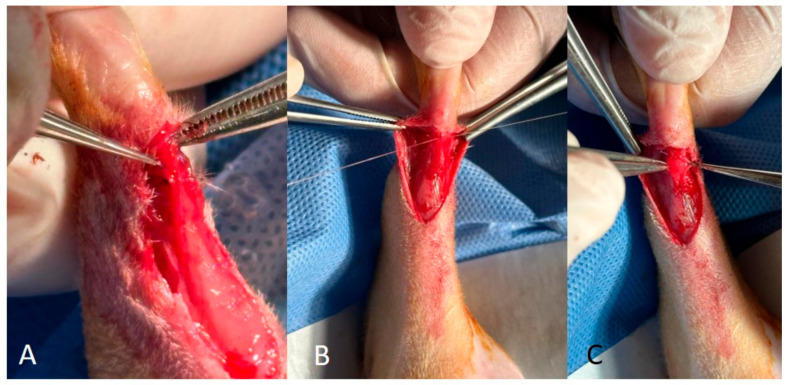
(**A**,**B**) Vascularised pedicle flap suturation and coverage. (**C**) Non-vascularised pedicle flap suturation and coverage.

In Group 2, the arterioles of the gastrocnemius fascia were identified (Figure 2A), the blood supply to the fascia was maintained, and a proximal-to-distal release of the gastrocnemius fascia was performed. Following this release, the Achilles tendon was transected transversely (Figure 2B). The Achilles tendon was repaired using a modified Kessler technique (Figure 2C), while the gastrocnemius fascia was attached to the Achilles tendon using epitendinous sutures with a vascularised pedicle (Figure 3A,B). Closure of the subcutaneous tissue and skin was orderly.

In Group 3, a similar proximal-to-distal release of the gastrocnemius fascia was performed. The distal end of the gastrocnemius fascia was then dissected to obtain a free graft. The Achilles tendon was cut transversely. The Achilles tendon was then repaired using a modified Kessler technique and the free gastrocnemius fascia was attached to the tendon using epitendinous sutures (Figure 3C). Closure of the subcutaneous tissue and skin followed in a systematic fashion.

### 2.3. Scintigraphy Test

**Figure 4:**
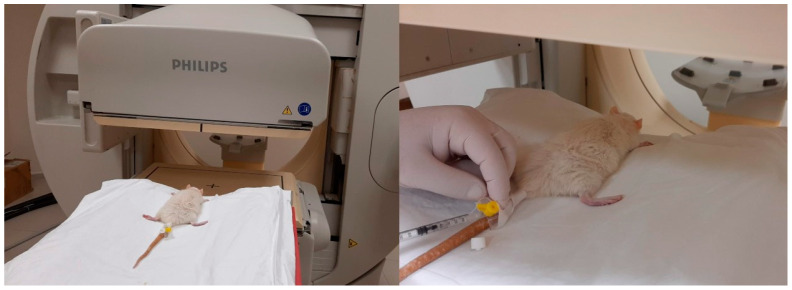
Tc-99m-methoxy isobutyl isonitrile (MIBI) was injected into the tail vein under a camera.

**Figure 5:**
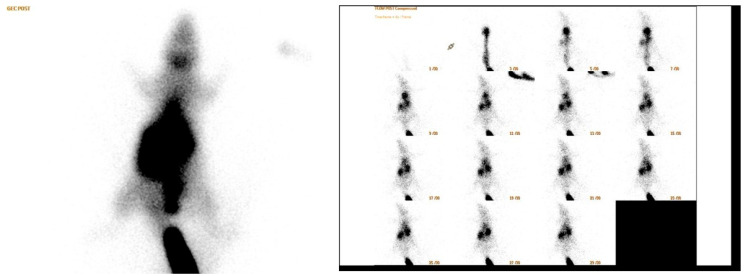
Scintigraphic images.

Anaesthesia was induced with ketamine (90 mg/kg body weight) and xylazine (10 mg/kg body weight) administered intraperitoneally. On postoperative day 14, rats were placed prone under a single-head SPECT/CT gamma camera (Philips Brightview XBT; Cleveland, OH, USA). A dose of 1 mCi (37 MBq) of Tc-99m-methoxyisobutyl isonitrile (MIBI) was injected into the tail vein of each rat under each camera (Figure 4). A low energy general purpose collimator (LEGP) was used for all scintigraphic imaging procedures. First, dynamic blood perfusion images were acquired in a 128 × 128 matrix in 1 min in 30 frames of 2 s, starting simultaneously with the injection. Then, early phase static blood pool images were acquired in a 256 × 256 matrix for 5 min after the dynamic images. Finally, the late static phase was acquired in a 256 × 256 matrix for 5 min 30 min after injection. All rats underwent surgery on the right leg. Therefore, all images were taken on the right side of the mark. After the images were obtained, a region of interest (ROI) of equal size was drawn on the surgical site at the location of the Achilles tendon and gastrocnemius muscle and on the contralateral leg of each rat in the early and late images. Counts in the ROI were calculated automatically. In addition, in the early and late static images, the total body count was calculated by drawing the ROI over the whole body of the rat. In this calculation, the activity remaining in the tail vein was excluded from the ROI. The images obtained were first evaluated visually by a specialist in nuclear medicine (Figure 5). Quantitative evaluation was performed using four parameters. The formulas for these parameters are as follows:

1. Perfusion reserve = (early static count on right ROI − late static count on right ROI)/early static count on right ROI;
2. R/L index = late ROI right/late ROI left.
3. Early count percentage = early static count on ROI right/early static count on ROI whole body
4. Late count percentage = late static count at right ROI/late static count at total body ROI

### 2.4. Histopathological Assessment

All rats were euthanised by decapitation at the end of day 21. Samples of 40 mm in length were taken from the Achilles tendon and the gastrocnemius muscle. At surgery, a piece of tendon (approximately 4 mm in cross section and 15 mm in length) was taken from the proximal posterocentral part of the bone tendon-bone graft. The distal part of the sample was first marked with a suture for orientation and later stained with India ink prior to histological processing. The tissue was then fixed in buffered saline and formalin and, as the specimens were small, dehydrated. All material was processed by dehydration in graded alcohols for 8 h, cleared in xylene, and embedded in paraffin. Sequential 4-pm sections were stained with haematoxylin and eosin (H&E), alcian blue at pH 2.5, and colloidal iron and examined by light microscopy, the H&E stains with and without polarisation. Tendons were evaluated for changes in tenocyte morphology, the presence or absence of ground substance, collagen bundle characteristics (using light and polarisation microscopy), and changes in vascularity.

At the end of day 21, changes in tenocyte morphology, ground substance presence, collagen bundle characteristics, and vascularity were assessed using a semiquantitative scoring system (Bonar scale). The same examiner randomly re-examined 50% of the histopathology slides. The scores for cellular, ground substance, collagen, and vascular changes from the first and second examinations were compared.

### 2.5. Statistical Analysis

In specimens where histopathological abnormalities were present we compared the semiquantitative score at the proximal pole (femoral attachment site) with a site 1 cm distally using the paired t-test. We compared the scores of abnormal and normal tendon using the unpaired t-test. The intraclass correlation coefficient (3. I) was used to examine the test retest reliability of the reevaluated tendons.

## 3. Results

In the primary repair group, one rat succumbed during the late phase of scintigraphy, while another rat died during anaesthesia administration prior to scintigraphy. As a result, scintigraphic data for the rat that died under anaesthesia were not obtained.

**Figure 6:**
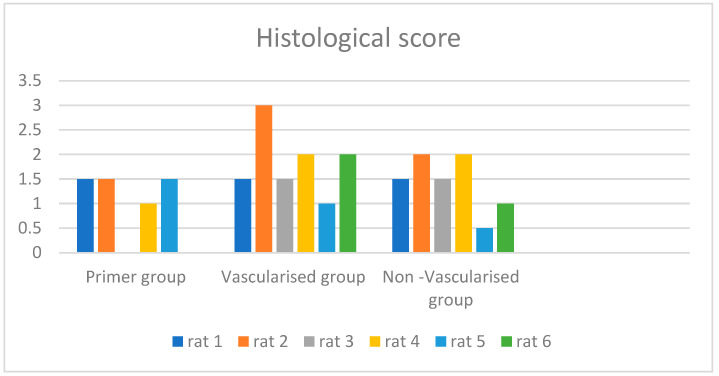
Histological score of groups.

The mean histopathological scores were as follows: 1.41 in both the primary repair group and the non-vascularised adipofascial flap group, and 1.83 in the vascularised pedicle group (Figure 6) (Table 1). Scintigraphic analysis revealed that the static late phase measurements were statistically significantly higher in the non-vascularised adipofascial flap group (*p* = 0.038). The mean late-phase R/L index was 1.77 in the non-vascularised adipofascial flap group, 1.51 in the vascularised pedicled adipofascial flap group, and 1.36 in the primary repair group. However, the difference between these groups was not statistically significant (*p* = 0.2). The mean perfusion reserve in the vascularised pedicled adipofascial flap group was 0.22, and in the non-vascularised adipofascial flap group it was 0.18. The difference between these groups was not statistically significant (*p* ≥ 0.05) (Table 1).

## 4. Discussion

The aim of the present study was to investigate the viability of vascularised and non-vascularised adipofascial flaps applied over a repaired tendon and tendon healing using a histological and scintigraphic rat model. The mean perfusion reserve was higher in the vascularised pedicled adipofascial flap group than in the non-vascularised adipofascial flap group. Blood perfusion was better in the vascularised pedicled adipofascial flap group. When the rats were sacrificed on day 21, the histopathological healing scores of the non-vascularised adipofascial flap and primary repair groups were the same at 1.41, while the vascularised pedicled adipofascial flap group had a score of 1.83. Koc et al. also found that tendon healing was better in the vascularised flap group in a biomechanical rat model [14]. According to these results, pedicled flaps did not adversely affect healing in the medium term. The mean R/L index in the late phase was 1.77 in the non-vascularised adipofascial flap group. Tendon grafts placed in scarred beds are less likely to perform optimally, as a non-adherent and unscarred vascular bed is required for smooth tendon gliding [5]. This study investigated the concept of interposing vascularised tissue, such as adipofascial flaps, between the tendon and surrounding tissue as a potential approach to improve tendon gliding and outcomes, particularly in injuries with a poor prognosis in the acute and subacute phases [6,7]. Scintigraphic measurements showed a statistically significant increase in the late static interpretation analysis in the non-vascularised adipofascial flap group (*p* = 0.038). These data also show that there is an increase in blood flow in the late period. Pedicle-free flaps can be used as a support tissue in tendon injuries where the blood supply to the flap could not be maintained. This study showed that in cases where the flap pedicle could not be preserved, the flaps were observed to be bleeding according to the scintigraphic results, so the pedicle-free flap can be used in cases where the tissue around the tendon is damaged. This result supports previous articles that claim free adipofascial flaps are beneficial in scar tissue with tenolysis [7]. Oztermeli et al. applied the fat graft over the Achilles repair area and found superior tenocyte value in the histopathological evaluation [15].

When using an adipofascial flap for tissue injury, if the vascular pedicle of the flap can be preserved, implantation of a pedicled flap may accelerate healing and may be the first choice. To date, many studies have been published using adipofascial flaps for defects in either the upper or lower extremity. In their review, Gir et al. identified a total of 186 lower extremity perforator pedicle flaps used for reconstruction, focusing primarily on the mid and distal thirds of the extremities. The types of pedicled perforator flaps reported were the peroneal artery perforator flap, posterior tibial artery perforator flap, anterior tibial artery perforator flap, medial sural artery perforator flap, lateral sural artery perforator flap, and lateral superior genicular artery perforator flap. The authors found that the peroneal artery perforator flap was the most commonly used flap, accounting for 47% of cases, followed by the posterior tibial artery perforator flap at 44%. They also reported that the most common causes of lower extremity defects were post-traumatic (38.8%) [16]. These findings suggest the importance of these two perforator flaps in lower extremity reconstruction, reflecting their reliability and versatility in addressing various defects. The literature has compared adipofascial flaps and fasciocutaneous flaps. Goil et al. compared adipofascial and two-stage fasciocutaneous reverse sural flaps in patients with lower leg trauma. They reported that the adipofascial reverse sural artery flap is a good option for patients with lower extremity trauma, with the added advantage of being a single-stage procedure with better donor site cosmesis compared to the fasciocutaneous reverse sural artery flap [17]. Li et al. conducted a study that focused on the use of distally based adipofascial sural flaps for complex foot and ankle wounds. The surgical approach involved elevating a distally-based lateral sural adipofascial flap, then inverting it and passing it from dorsal to plantar to address the deep dead space within the wound. The authors highlighted several advantages associated with the adipofascial flap technique. Of particular note was its ability to provide superior cosmetic results while minimising donor site morbidity. This was primarily attributed to the ability to close the margins directly without tension, thus reducing the risk of wound complications. Emphasis was placed on preserving the subdermal plexus during flap elevation, a critical factor in preventing flap edge necrosis. Furthermore, the inclusion of a wide perforator plus adipofascial pedicle was emphasised to ensure robust vascularisation of the flap, thereby increasing its viability and overall success [1].

Schmidt et al. reported that the simple modification of the distal sural artery flap as an adipofascial flap is easier and faster to perform than the fasciocutaneous flap. In addition, the adipofascial flap technique did not require an island of skin, which speeds up the procedure. The main advantage was the primary closure of the donor site and the harvesting of a thin, pliable flap [18]. These studies collectively address various aspects and applications of adipofascial flaps and other reconstructive techniques in the treatment of complex wounds in the distal leg, foot, and ankle. Further research is warranted to investigate the effects of these techniques on tissue healing, functional recovery, and aesthetic outcomes.

Losco et al. published an article using the reverse adipofascial flap for fingertip reconstruction. They reported that the modified single-pedicle reverse adipofascial flap for fingertip coverage is a reliable and effective method, and that the increased degrees of rotation at the pivot point offer a viable solution to several complicated surgical obstacles encountered in fingertip reconstruction procedures [19]. Laoulakos et al. also proposed adipofascial flaps for fingertip amputation reconstruction [20]. Idone et al. modified the adipofascial flaps by fenestrating the flap and restoring the nail bed [21]. Facchin et al. used a multidorsal metacarpal artery perforator adipofascial turnover flap for index to little finger reconstruction [22].

Technetium-99m methoxyisobutyl isonitrile (Tc-99m MIBI, Sestamibi) is a lipophilic monovalent cation mainly used in myocardial perfusion studies. Due to the negative and lipid structure of the membrane potential, it passes through plasma and mitochondrial membranes by passive diffusion and is retained in mitochondria to a significant extent within the cell. In addition, it is an effective agent used in limb perfusion and viability by showing involvement in skeletal muscle. Cellular uptake and trapping of 99mTc-sestamibi is related not only to regional blood flow but also to mitochondrial metabolic conditions and viability [23]. The retention of Tc-99m-MIBI in mitochondria also allows late imaging. The persistence of MIBI uptake in delayed images may indicate tissue viability. Technetium-99m sestamibi (99mTc-MIBI) delayed phase imaging has been widely used to diagnose myocardial ischaemia [24]. However, one study reported that mitochondrial retention of Tc-99m-MIBI was not organ specific and showed the same pattern of involvement in many tissues such as skeletal muscle [25].

The limitation of this study was that the gait analysis or walking speed of the rats for functional assessment could not be obtained. Tendon healing was analysed in the histological assessment, but tendon adhesion could not be measured.

## 5. Conclusions

The increased late involvement of non-pedicled flaps on scintigraphy images was interpreted as an indication of flap bleeding, while the higher histopathological scores in the pedicled flap group suggest that pedicled flaps may promote faster healing. Based on these findings, we cautiously recommend the use of pedicled flaps when early tendon mobilisation is required, provided the pedicle is preserved. However, these results should be interpreted with caution before drawing broad clinical implications, as this study was conducted in a controlled experimental rat model.

Further research is needed to validate these findings in larger animal models and ultimately in the human clinical setting. Future studies should focus on long-term functional outcomes, the optimal timing for initiating tendon mobilisation, and the effects of different flap techniques on tendon strength and healing quality. Investigating the role of vascularisation and other biological factors in the healing process may also provide valuable insights into improving tendon repair strategies.

## Figures and Tables

**Table 1:** Histological scores, R/L index, perfusion reserve, and static late discussion of groups.

Rat	Histological Score	Late R/L İndex	Perfusion Reserve	Static Late Discussion
Primer group rat 1	1.5	1.77	0.3	4
Primer group rat 2	1.5	1.43	0.25	2
Primer group rat 3				
Primer group rat 4	1	1.02	0.01	1
Primer group rat 5	1.5	1.23	0.12	3
Primer group rat 6				
Vascularised group rat 1	1.5	1.57	0.34	3
Vascularised group rat 2	3	1.45	0.19	1
Vascularised group rat 3	1.5	1.74	0.1	2
Vascularised group rat 4	2	1.88	0.29	3
Vascularised group rat 5	1	1.16	0.31	1
Vascularised group rat 6	2	1.28	0.08	2
Non-Vascularised group rat 1	1.5	1.52	0.19	4
Non-Vascularised group rat 2	2	1.62	0.15	1
Non-Vascularised group rat 3	1.5	1.76	0.45	4
Non-Vascularised group rat 4	2	2.66	0.03	4
Non-Vascularised group rat 5	0.5	1.15	0.24	3
Non-Vascularised group rat 6	1	1.91	0.06	3

## Data Availability

The datasets used and/or analyzed during the current study are available from the corresponding author upon reasonable request.
